# Physical Health Indicators Improve Prediction of Cardiovascular and All-cause Mortality among Middle-Aged and Older People: a National Population-based Study

**DOI:** 10.1038/srep40427

**Published:** 2017-01-12

**Authors:** Wei-Ju Lee, Li-Ning Peng, Shu-Ti Chiou, Liang-Kung Chen

**Affiliations:** 1 Aging and Health Research Center, National Yang Ming University, Taipei, Taiwan; 2 Institute of Public Health, National Yang Ming University, Taipei, Taiwan; 3 Department of Family Medicine, Taipei Veterans General Hospital Yuanshan Branch, Taipei, Taiwan; 4 Center for Geriatrics and Gerontology, Taipei Veterans General Hospital, Taipei, Taiwan; 5 Health Promotion Administration, Ministry of Health and Welfare, R.O.C., Taiwan

## Abstract

The effectiveness of established methods for stratifying cardiovascular risk, for example, the Framingham risk score (FRS), may be improved by adding extra variables. This study evaluated the potential benefits of adding physical health indicators (handgrip strength, walking speed, and peak expiratory flow) to the FRS in predicting cardiovascular and all-cause mortality by using a nationwide population-based cohort study data. During median follow-up of 4.1 years, 67 of 911 study subjects had died. In Cox regression analysis, all additional physical health indicators, except walking speed, significantly predicted cardiovascular and all-cause mortality (*P* < 0.05). Compared with the conventional FRS, *c* statisti*c*s were significantly increased when dominant handgrip strength or relative handgrip strength (handgrip strength adjusted for body mass index), or combination with walking speed or peak expiratory flow were incorporated into the FRS prediction model, both in the whole cohort and also in participants who did not have prevalent cardiovascular diseases at baseline. In conclusion, dominant or relative handgrip strength are simple and inexpensive physical health indicators that substantially improve the accuracy of the FRS in predicting cardiovascular and all-cause mortality among middle-aged and older people.

Despite the best efforts of modern health care services to detect and manage cardiovascular diseases, their burden in terms of disability-adjusted life years remains undoubtedly high; this may be the result of global population aging and westernization of lifestyle^1^,^2^. It is important for policymakers and healthcare professionals to refine ways to assess cardiovascular risk in the general population^3^, to stratify people into different risk categories, and to implement preventive intervention programs for those with a high degree of cardiovascular risk^4^.

The Framingham risk score (FRS) is a widely used cardiovascular risk-assessment tool that is based on conventional cardiovascular risk factors, which include age, sex, smoking status, systolic blood pressure, total cholesterol and high-density lipoprotein cholesterol (HDL-C); the FRS computes scores based on the presence of these risk factors to estimate 10-year risk of cardiovascular events^5^. Although the FRS has been applied extensively in both clinical practice and research, it is not fully reliable and the addition of certain new biomarkers has been found to improve its accuracy^6^,^7^. A variety of novel biomarkers and physical measurements have been reported to be associated with the risk of cardiovascular diseases^8^,^9^,^10^,^11^,^12^, and these may be candidate indicators to improve the effectiveness of the FRS. However, most previous studies have used odds ratios rather than C-statistics, which reflect the area under the receiver operating characteristic curve (ROC), to ascertain associations between variables and mortality^7^,^11^,^13^, which may be an injudicious statistical approach; specifically, it has been propounded that single measures of association, such as odds ratios, do not meaningfully confer the ability to classify subjects into lower or higher risk categories. Conversely, the C-statistic may afford significantly better stratification ability^14^,^15^.

Physical health indicators are simpler, cheaper and easier to use than serum biomarkers – advantages that would be of great value in large-scale community studies, daily clinical practice and public health programs. Despite evidence that walking speed^16^, handgrip strength^12^, relative handgrip strength (relative handgrip strength adjusted by body mass index)^17^ and peak expiratory flow rate^18^ all predict cardiovascular and all-cause mortality, the added clinical benefits of incorporating such physical health indicators into conventional risk estimation models remains uncertain^19^. High-sensitivity C-reactive protein (hs-CRP) has previously been associated with cardiovascular mortality^6^,^13^, but the value on routine screening of general population is uncertain^20^. It is of particular interest to know whether adding hs-CRP and the aforementioned physical metrics may provide prognostic information beyond FRS that is salient to the risk of cardiovascular death. Therefore, this national study aimed to investigate whether incorporating hs-CRP, handgrip strength, relative handgrip strength, walking speed, and peak expiratory flow rate into the FRS algorithm would improve its accuracy in predicting the risk of cardiovascular death as well as all-cause mortality.

## Results

Table 1 summarizes the characteristics of the whole study cohort and compares differences between men and women. The youngest subject was 53 and the oldest was 85 years old. Median follow-up was 4.1 years, during which 67 participants died (1.8 per 100 person-years at risk), with 20 deaths attributable to cardiovascular disease (0.5 per 100 person-years at risk). Among 748 participants without cardiovascular disease at baseline, 46 died (1.2 per 100 person-years at risk), 11 from cardiovascular disease (0.3 per 100 person-years at risk). There was no participant loss to follow-up in the survival analysis.

Table 2 shows ROC-determined optimal cut-off points for stratifying the risk of mortality in this cohort. Handgrip strength and relative handgrip strength had bigger C-statistics than walking speed, peak flow, or hs-CRP. In this study, the cut-off points for relative handgrip strength of 1.40 in women and 2.34 in men had the best discrimination ability, with sensitivity of 80.0% and 70.2% and specificity of 67.4% and 74.2%, respectively.

### Cox regression

Age and sex adjusted associations of FRS, hs-CRP, dominant handgrip strength, relative handgrip strength, and peak flow rate, with cardiovascular and total mortality were all significant (Table 3). Multivariate Cox regression analysis showed that FRS, hs-CRP, dominant handgrip strength, relative handgrip strength, and peak flow rate were all significant predictors for cardiovascular and all-cause mortality after 4-year follow-up for the whole sample, as well as the participants without cardiovascular disease at baseline (Table 4). Although walking speed was associated with cardiovascular and all-cause mortality in the whole sample and among those without prevalent cardiovascular disease, this association became insignificant after adjusting for age and sex.

### Discrimination

Incremental C-statistics were examined when hs-CRP and physical health metrics were added to the conventional FRS model: either handgrip strength or relative handgrip strength improved prediction of all-cause mortality in the whole sample and in subjects without prevalent cardiovascular diseases (Table 5). Adding walking speed or peak expiratory flow did not significantly improve FRS predictions for cardiovascular and all-cause mortality; however, either dominant or relative handgrip strength combined with walking speed or peak expiratory flow had higher C-statistics, indicating higher discrimination ability for risk of death.

## Discussion

This national population-based cohort study showed significantly improved prediction of all-cause mortality when hs-CRP, dominant or relative handgrip strength were added to the established FRS model. C-statistics for cardiovascular mortality increased when dominant or relative handgrip strength combined with walking speed or peak flow rate were added. These improvements still remained strong even when participants with cardiovascular disease at baseline were excluded; furthermore, these associations remained consistent in three alternative models, with metrics input as continuous or categorized variables, defined according to previous reports, or with optimal cut-offs defined by ROC analysis of this cohort. The overall results not only affirm previous reports^11^,^12^,^17^,^18^,^21^,^22^,^23^, but show in addition that adding handgrip strength and/or peak flow to the FRS algorithm would improve cardiovascular mortality and all-cause death, which is a novel finding.

Walking speed was associated with mortality and predicted 30-day mortality in patients receiving transcatheter valve therapy^16^,^24^. Although walking speed in this study was inversely associated with cardiovascular mortality and all-cause death in the univariate analysis (Table 3), unlike previous studies^11^,^16^ this correlation became insignificant after adjusting for potential confounders. There are two possible reasons that walking speed did not improve mortality prediction in this study: first, the participants were 10–12 years younger than those in previous reports, which may diminish the accuracy of mortality prediction; second, the mortality risk estimated by walking speed obtained over different distances is known to vary greatly. For example, the risk may be overestimated by using 10-metre walking speed, but underestimated by using 4-metre walking speed^25^,^26^. This study assessed walking speed over 3-metres.

Hs-CRP was independently associated with cardiovascular and all-cause mortality in the whole cohort and those without prevalent cardiovascular diseases, consistent with previous reports^6^,^13^,^27^. However, the C-statistic increment when adding hs-CRP to FRS did not reach statistical significance in predicting cardiovascular death, consistent with recommendations of the ACC/AHA that hs-CRP assessment should be reserved for those with uncertain risk defined by established qualitative methods (class IIb)^19^, and of the US Preventive Service Task Force not to use hs-CRP for routine screening of the general population^20^.

Handgrip strength has previously been associated with mortality^11^,^12^; however, some have argued that body size would confound the correlation of handgrip strength with cardiovascular health, and proposed to incorporate body size into muscle strength^28^,^29^. The Foundation of the United States National Institute of Health has proposed a new index to correct for body size, which divides muscle strength by BMI^30^. Similarly, relative handgrip strength, defined as the totaled handgrip strength of both hands, divided by BMI, was applied to represent muscle strength. Previous reports found that relative handgrip strength was associated with cardiometabolic risk^28^,^31^, and predicted mobility limitation better than dominant handgrip strength^32^. Results from the study showed that dominant and relative grip strength had very similar predictive values for mortality, which implied that it is appropriate to use the simpler measure i.e. dominant grip strength.

Peak expiratory flow is easily measured using a hand-held device in community settings; it is a proxy for the strength of respiratory muscle, and has good prognostic value for mortality and disability^18^,^33^. In a study of 170000 Chinese men, the association between peak flow and cardiovascular mortality attenuated gradually over 15 years^18^, congruent with previous findings^18^,^33^. In this study, incorporating peak expiratory flow to FRS combined with relative or dominant handgrip strength significantly improved the prediction of cardiovascular and all-cause mortality.

The C-statistic is a popular discriminatory metric that distinguishes those with outcomes of interest from those without. The significantly increased C-statistics from this study suggest that adding handgrip strength and peak flow would substantially improve the FRS. However, some researchers have questioned sole reliance on the C-statistic to determine the effectiveness of a new indicator because a significant C-statistic increment may require a large degree of independent association^15^,^34^. The fact that the results of this study remained consistent through all analytic models, subgroup analyses, and statistical analyses, strongly supports the veracity of the findings presented here.

This study has important public health implications. First, adding physical health indicators to the FRS significantly improved risk stratification for cardiovascular and all-cause mortality. Moreover, measurements such as handgrip strength and peak flow are straightforward, convenient, inexpensive, and reliable, which are ideal features of tools used for routine assessments in community studies and clinical practice to better stratify cardiovascular risk. Second, a recent meta-analysis of 28 randomized controlled trials suggested that resistance-training of handgrip strength would improve cardiovascular risk^35^; it implied that large-scale screening for muscle strength and providing resistance-training programs would greatly benefit overall cardiovascular health. Another advantage of physical indicators is easy comprehension by the general public, and participants would be encouraged if their performance were gradually improved after the program. Third, norms of physical health metrics may differ according to ethnic, socio-economic and cultural backgrounds. Optimal cut-off points of walking speed, dominant and relative handgrip strength and peak flow for mortality prediction provided by this nationally-representative cohort would allow local policymakers and healthcare professionals to mobilize and prioritize resources for those at higher risk.

Despite our best efforts this study still had limitations. First, the results of this predominantly Chinese national cohort from Taiwan may not be completely applicable to populations with different ethnic backgrounds. Second, diabetes mellitus, hypertension and other comorbidities were self-reported, rather than extracted from medical records; however, the interviewers did emphasize that self-reported diagnoses should have been made by a physician, which would minimize this potential bias. Third, sex-specific analysis was not performed due to the relatively limited sample size. Fourthly, the exclusion of those with missing data inevitably affected the representativeness of the cohort and the generalizability of the findings; such individuals had lower socioeconomic status in terms of the MacArthur scale of subjective economic status (4.1 ± 2.0 versus 4.4 ± 1.9, p < 0.001)^36^ and had a higher mortality rate (19.3% versus 7.4%, p < 0.001).

## Conclusion

Dominant or relative handgrip strength are accurate, inexpensive and simple physical health indicators that substantially improved FRS risk stratification for cardiovascular and all-cause mortality among community-dwelling middle-aged and older people; these metrics also have considerable potential for evaluating the effectiveness of intervention programs.

## Methods

### Study population

This national population-based cohort study data extracted from the second wave of the Social Environment and Biomarkers of Aging Study in Taiwan, using multistage proportional-to-size sampling strategies to ensure that these were nationally representative; details of the study design, participant recruitment, and data collection procedures are already published^37^. Briefly, 1284 participants were interviewed face-to-face at home, and 1036 subsequently underwent serum biochemistry tests and physical examinations: 911 completed comprehensive physical health assessments of handgrip strength, relative handgrip strength, walking speed, and peak expiratory flow, besides having laboratory results of hs-CRP and other covariates analyzed in this study. Among these, data on 748 participants without prevalent cardiovascular diseases (self-reported physician diagnosis) at baseline were extracted for further analysis. A written informed consent was obtained from every participant. The Joint Institutional Review Board of Taiwan approved the study protocol. The design and procedures of the study were carried out in accordance with the principles of the Declaration of Helsinki.

### Baseline examinations

All participants were invited to nearby hospitals for anthropometric measurements, physical function assessments and venous blood sampling after an overnight fast. Serum hs-CRP levels were measured by immunoturbidimetry (Roche Cobas Integra800, Basel, Switzerland), with sensitivity of 0.71 mg/l and 2.5% inter-assay coefficient of variance. Serum levels of total cholesterol and HDL-C were measured using commercial kits (Beckman Coulter Synchron LX20 Inc., Fullerton, CA, USA), which both had sensitivity of 5 mg/dl and respective inter-assay coefficients of variance of 1.4% and 3.1%.

Height, body weight and blood pressure were measured by standard procedures. Body mass index (BMI) was calculated as body weight in kilograms, divided by height in meters squared; the average of three successive blood pressure readings by an automatic monitor (Omron^®^ Model HEM-7011, Kyoto, Japan) was used as the blood pressure for further analysis. The North Coast™ hydraulic hand-dynamometer (NC70142, California, US) was used to measure isometric handgrip strength: research nurses adjusted the dynamometer according to the palm size of each individual; participants remained seated with the dynamometer held perpendicular to their flexed elbow; maximal readings of three measurements from each hand were recorded. Dominant grip strength was defined as a maximum reading of three dominant hand measurements. Relative handgrip strength was defined as totaled maximal handgrip strengths of both hands, divided by BMI^31^. Walking speed at ordinary pace was measured by a 3-metre walking test, performed from a static start and without deceleration, according to the interviewer’s stopwatch. Peak flow rate was measured by a TruZone peak flow meter(Trudell Medical, Ontario, Canada) with participants standing: peak flow was defined as the maximum of three readings of expiration at maximal force that began from maximal lung inflation^38^.

### Outcomes and follow-up

The cause of death for each deceased participant was identified from the Taiwan national death registry; the dependent variable of interest was defined as participants who died between their original interview and 31 December 2010. The 10^th^ version of International Classification of Disease (ICD-10) codes I00 to I99, or 9^th^ version of International Classification of Disease Clinical Modification (ICD-9 CM) codes 390 to 459 were denoted as cardiovascular disease.

### Statistical analysis

Numerical variables were expressed as means plus/minus standard deviation and categorical variables were expressed as proportions. Since the distribution of serum hs-CRP levels was skewed, these data were logarithmically transformed to achieve a normal distribution. Cox proportional hazard regression was used to evaluate the association between mortality and hs-CRP, handgrip strength, relative handgrip strength, walking speed, and peak flow. Age and sex adjusted rates and models adjusted for established risk factors, including age, sex, systolic blood pressure, antihypertensive treatment, total cholesterol, HDL-C, lipid-lowering treatment, diabetes, smoking status, and BMI, were analyzed. Multivariate Cox regression used three models: First, factors predicting mortality were examined by continuous variables, in terms of increment per standard deviation, which was easy to compare between different units of various measurements. Next, continuous measurements were assigned to higher or lower groups according to both published definitions (hs-CRP, >3.0 mg/l^21^; handgrip strength, <14.3 kg for women and <22.4 kg for men^39^; relative handgrip strength, <0.79 kg/BMI for women and <1.32 kg/BMI for men^32^; walking speed, <0.8 m/s^25^,^40^; peak expiratory flow rate, <250.0 l/min)^18^, and cut-off values that achieved optimal discrimination by ROC analysis (hs-CRP, >3.0 mg/l; handgrip strength, <16.0 kg for women and <28.0 kg for men; relative handgrip strength, <1.40 kg/BMI for women and <2.34 kg/BMI for men; walking speed, <0.8 m/s; peak expiratory flow rate, <250.0 l/min). The ROC curve is plotted as sensitivity, also known as true positive rate, against (1 minus specificity), also known as false positive rate, at all possible threshold settings. Youden’s index, a main ROC summary statistic used to determine the optimal cut-off points to obtain the greatest effectiveness of the variables, was calculated as (sensitivity plus specificity minus 1)^41^; Youden’s index maxima were selected as optimal cut-off values. ROC analysis was used to calculate FRS C-statistics for hs-CRP, handgrip strength, relative handgrip strength, walking speed and peak flow rate. Differences in C-statistics after adding the aforementioned items into a model with established risk factors were estimated according to the method of DeLong *et al*.^42^. All models were run on all-cause mortality and then repeated, by using a competing risks framework, to examine CVD mortality.

A *P*-value < 0.05 from two-sided tests was considered statistically significant. All analyses were performed with the SAS statistical package, version 9.4 (SAS Institute, Inc., Cary, NC, USA).

## Additional Information

**How to cite this article**: Lee, W.-J. *et al*. Physical Health Indicators Improve Prediction of Cardiovascular and All-cause Mortality among Middle-Aged and Older People: a National Population-based Study. *Sci. Rep.* **7**, 40427; doi: 10.1038/srep40427 (2017).

**Publisher's note:** Springer Nature remains neutral with regard to jurisdictional claims in published maps and institutional affiliations.

## Figures and Tables

**Table 1:** Baseline characteristics of all participants and sex-specific comparison.

Characteristic: data values show mean ± standard deviation, or number (%)	Total	Men	Women	p value
Total numbers	911	504	407	
Age (years)	65.3 ± 9.3	66.2 ± 9.5	64.2 ± 8.9	0.001
Body mass index (kg/m^2^)	24.8 ± 3.4	24.6 ± 3.1	24.9 ± 3.7	0.159
Fasting blood glucose (mg/dl)	106.7 ± 30.6	107.7 ± 31.4	105.6 ± 29.5	0.291
Serum cholesterol (mg/dl)				
Total	198.8 ± 37.9	192.5 ± 37.1	206.6 ± 37.4	< 0.001
HDL	47.9 ± 14.0	44.8 ± 12.9	51.8 ± 14.2	< 0.001
Blood pressure (mmHg)				
Systolic	139.1 ± 20.3	140.3 ± 20.0	137.8 ± 20.7	0.064
Diastolic	79.1 ± 11.6	80.5 ± 12.2	77.3 ± 10.7	< 0.001
Framingham risk score	12.3 ± 9.1	17.4 ± 7.7	6.0 ± 6.3	< 0.001
High-sensitivity C-reactive protein (mg/l)	2.6 ± 5.7	0.3 ± 0.6	0.2 ± 0.5	0.188
Dominant handgrip strength (kg)	27.7 ± 10.2	33.8 ± 8.7	20.1 ± 6.1	< 0.001
Relative handgrip strength (kg/BMI)	2.2 ± 0.8	2.7 ± 0.7	1.6 ± 0.5	< 0.001
Walking speed (m/s)	0.9 ± 0.3	0.9 ± 0.3	0.8 ± 0.3	< 0.001
Peak flow (l/min)	343.4 ± 138.1	406.8 ± 139.7	265.0 ± 86.2	<0.001
Smoker	178 (19.5%)	10 (2.5)	168 (33.3)	<0.001
Diabetes	141 (15.5%)	70 (17.2)	71 (14.1)	0.197
Hypertension	288 (31.6%)	127 (31.2)	161 (31.9)	0.811
Antihypertensive treatment	272 (29.9%)	123 (30.2)	149 (29.6)	0.829
Lipid-lowering treatment	60 (6.6%)	26 (6.4)	34 (6.8)	0.829
Previous cardiovascular disease	163 (17.9%)	71 (17.4)	92 (18.3)	0.751

HDL, high-density lipoprotein; BMI, body mass index.

**Table 2:** Area under curve and cut-off points of physical metrics.

Variable	AUC (95% CI)	Cut-off	Sensitivity (%)	Specificity (%)
High-sensitivity CRP	0.65 (0.58–0.72)	3.0	43.3	81.3
Dominant grip strength				
Women	0.74 (0.63–0.85)	16.0	70.0	73.6
Men	0.76 (0.69–0.84)	28.0	68.1	75.5
Relative grip strength				
Women	0.77 (0.68–0.86)	1.40	80.0	67.4
Men	0.76 (0.68–0.83)	2.34	70.2	74.2
Walking speed	0.73 (0.65–0.81)	0.8	68.7	55.5
Peak expiratory flow	0.66 (0.59–0.73)	250.0	53.7	72.9

AUC, area under curve; CI, confidence interval; CRP, C-reactive protein.

**Table 3:** Age and sex adjusted associations of established risk factors, hs-CRP, and physical indicators with cardiovascular and all-cause mortality.

	Cardiovascular mortality				All-cause mortality			
Variables	Hazard ratio	95% CI	C-statistics	*P-value*	Hazard ratio	95% CI	C-statistics	*P-value*
**Basic model**								
Systolic blood pressure per SD change	1.8	1.2–2.7	0.77	<0.001	1.2	0.9–1.5	0.74	0.210
Antihypertensive treatment	1.4	0.6–3.6	0.73	0.459	1.1	0.6–1.9	0.74	0.777
Total cholesterol per SD change	0.9	0.6–1.4	0.74	0.601	1.0	0.8–1.3	0.74	0.909
HDL-cholesterol per SD change	0.7	0.4–1.3	0.73	0.286	0.8	0.5–1.0	0.73	0.007
Lipid-lowering treatment	2.3	0.6–8.3	0.73	0.202	1.9	0.8–4.3	0.74	0.136
Diabetes	2.0	0.7–5.7	0.74	0.195	1.9	1.0–3.6	0.74	0.038
Smoking	0.9	0.3–2.9	0.73	0.856	0.8	0.4–1.6	0.74	0.533
Body mass index per SD change	1.1	0.7–1.8	0.74	0.597	1.1	0.8–1.4	0.74	0.568
**Predictive parameter**								
Framingham risk score per SD change	2.4	1.3–4.5	0.78	0.006	1.3	0.9–1.9	0.74	0.157
High-sensitivity CRP per SD change	1.6	1.2–2.2	0.77	0.004	1.6	1.3–1.9	0.77	<0.001
Dominant handgrip strength per SD change	0.5	0.3–0.9	0.77	0.012	0.4	0.3–0.5	0.78	<0.001
Relative handgrip strength per SD change	0.5	0.3–0.8	0.79	0.010	0.4	0.3–0.6	0.79	<0.001
Walking speed per SD change	0.7	0.4–1.1	0.74	0.132	0.8	0.6–1.0	0.74	<0.083
Peak flow rate per SD change	0.5	0.2–0.9	0.78	0.016	0.5	0.4–0.7	0.76	<0.001

CI, confidence interval; HDL, high-density lipoprotein; CRP, C-reactive protein.

**Table 4:** Hazard ratios for death from all-cause and cardiovascular causes according to physical indicators*.

Indicators	Entire cohort				Participants without CVD at baseline			
	Cardiovascular death		All-cause death		Cardiovascular death		All-cause death	
	95% CI	*P-*value	95% CI	*P-*value	95% CI	*P*-value	95% CI	*P-*value
Framingham risk score								
1-SD increase	2.7 (1.7–4.4)	<0.001	1.9 (1.5–2.3)	<0.001	1.9 (1.1–3.4)	0.024	1.6 (1.3–2.2)	<0.001
<10%	Reference		Reference					
10%–20%	2.5 (0.6–10.4)	0.213	1.7 (0.9–3.3)	0.130	1.7 (0.3–8.3)	0.529	2.0 (0.9–4.2)	0.086
≥0%	6.5 (1.8–23.1)	0.004	3.4 (1.9–6.1)	<0.001	3.0 (0.7–12.7)	0.128	3.1 (1.5–6.3)	0.002
High-sensitivity CRP^†^								
1-SD increase	1.7 (1.2–2.3)	0.001	1.5 (1.3–1.8)	<0.001	1.7 (1.2–2.5)	0.007	1.4 (1.2–1.7)	0.001
>3.0 mg/l^‡§^	3.4 (1.4–8.3)	0.008	2.6 (1.6–4.3)	<0.001	3.0 (0.9–10.2)	0.077	2.1 (1.1–3.8)	0.02
Dominant handgrip strength^†^								
1-SD increase	0.4 (0.2–0.8)	0.004	0.4 (0.3–0.6)	<0.001	0.4 (0.2–0.8)	0.007	0.4 (0.3–0.6)	<0.001
<14.3 kg women, <22.4 kg men^‡^	4.3 (1.6–11.3)	0.004	3.8 (2.2–6.5)	<0.001	7.3 (2.0–26.8)	0.003	5.2 (2.7–10.0)	<0.001
<16.0 kg women <28.0 kg men^§^	4.2 (1.5–11.5)	0.006	3.1 (1.8–5.4)	<0.0001	8.5 (2.2–32.2)	0.002	4.1 (2.1–7.9)	<0.0001
Relative handgrip strength*								
1-SD increase	0.5 (0.3–0.8)	0.006	0.5 (0.3–0.6)	<0.001	0.4 (0.2–0.8)	0.006	0.4 (0.3–0.6)	<0.001
<0.79 women, <1.32 men^‡^	1.1 (0.1–8.6)	0.914	2.3 (1.0–5.2)	0.050	0.0 (0.0–NR)	0.995	2.4(0.8–7.1)	0.121
<1.40 women, <2.34 men^§^	3.4 (1.2–9.5)	0.017	3.9 (2.2–7.0)	<0.0001	6.3 (1.5–26.1)	0.011	4.6(2.3–9.2)	<0.0001
Walking speed^†^								
1-SD increase	0.7 (0.4–1.2)	0.179	0.8 (0.6–1.1)	0.110	0.7 (0.3–1.5)	0.313	0.8(0.6–1.2)	0.293
<0.8 m/s^‡,§^	1.0 (0.3–2.7)	0.925	1.2 (0.7–2.1)	0.526	0.8 (0.2–3.3)	0.734	1.3(0.6–2.5)	0.526
Peak expiratory flow^†^								
1-SD increase	0.4 (0.2–0.8)	0.011	0.5 (0.4–0.7)	<0.001	0.4 (0.2–0.9)	0.037	0.5(0.4–0.8)	0.003
<250.0 l/min^‡,§^	3.2 (1.2–8.5)	0.023	2.7 (1.6–4.7)	0.0003	3.3 (0.8–13.5)	0.104	2.6(1.3–5.1)	0.005

CVD, cardiovascular disease; CI, confidence interval; SD, standard deviation; CRP, C-reactive protein; NR, not reached.

*Results were calculated by Cox regression analysis. Data were adjusted for: age at baseline (continuous); sex (binary); systolic blood pressure (continuous); use or nonuse of antihypertensive medication (binary); total cholesterol (continuous); high-density lipoprotein cholesterol (continuous); use or nonuse of lipid lowering medication (binary); presence or absence of diabetes (binary); smoking status (binary).

^†^Data were adjusted for variables listed in footnote* plus body mass index (continuous).

^‡^Cut-off values according to previous literature.

^§^Cut-off values according to this study.

**Table 5:** Comparison of discrimination for cardiovascular and all-cause mortality when one, two or three physical health indicators are added to a model including age, sex and the Framingham risk score.

Indicators	Entire cohort C-statistics (95% CI) for				Participants without CVD at baseline C-statistics (95% CI for:			
	Cardiovascular death	*P-*value*	All-cause death	*P-*value*	Cardiovascular death	*P-*value*	All-cause death	*P-*value*
Framingham risk score	0.78(0.66–0.89)	Reference	0.74(0.68–0.80)	Reference	0.70(0.50–0.90)	Reference	0.72(0.65–0.80)	Reference
**Single indicator**								
High-sensitivity CRP	0.83(0.73–0.92)	0.188	0.77(0.71–0.83)	0.007	0.82(0.69–0.94)	0.250	0.75(0.68–0.82)	0.256
Dominant handgrip	0.81(0.71–0.91)	0.310	0.78(0.72–0.84)	0.039	0.83(0.75–0.92)	0.076	0.77(0.70–0.85)	0.068
Relative handgrip	0.81(0.72–0.90)	0.123	0.79(0.73–0.84)	0.008	0.82(0.72–0.91)	0.066	0.78(0.71–0.85)	0.043
Walking speed	0.77(0.66–0.89)	0.787	0.74(0.68–0.80)	0.952	0.71(0.52–0.90)	0.640	0.72(0.65–0.80)	0.705
Peak expiratory flow	0.81(0.71–0.91)	0.157	0.76(0.69–0.82)	0.170	0.80(0.67–0.92)	0.171	0.74(0.66–0.82)	0.491
**Two indicators**								
Dominant handgrip + walking speed	0.76(0.71–0.82)	0.008	0.78(0.72–0.84)	0.032	0.73(0.66–0.80)	0.006	0.77(0.70–0.85)	0.068
Dominant handgrip + peak expiratory flow	0.77(0.71–0.83)	0.021	0.79(0.73–0.85)	0.0271	0.73(0.65–0.81)	0.011	0.78(0.70–0.86)	0.075
Relative handgrip + walking speed	0.77(0.71–0.82)	0.002	0.79(0.73–0.84)	0.008	0.72(0.65–0.80)	0.008	0.78(0.71–0.85)	0.043
Relative handgrip + peak expiratory flow	0.77(0.72–0.83)	0.004	0.80(0.74–0.85)	0.006	0.73(0.65–0.81)	0.008	0.78(0.71–0.86)	0.031
Walking speed + peak expiratory flow	0.74(0.68–0.80)	0.146	0.76(0.69–0.82)	0.1569	0.67(0.59–0.76)	0.110	0.74(0.66–0.82)	0.460
**Three indicators**								
Dominant handgrip + walking speed + peak expiratory flow	0.77(0.71–0.83)	0.018	0.79(0.73–0.85)	0.031	0.73(0.65–0.81)	0.011	0.78(0.70–0.86)	0.083
Relative handgrip + walking speed + peak expiratory flow	0.77(0.72–0.83)	0.004	0.80(0.74–0.85)	0.007	0.73(0.65–0.81)	0.008	0.78(0.71–0.86)	0.034

CI, confidence interval; CVD, cardiovascular disease; CRP, C-reactive protein.

*All models were adjusted for age and sex. P values are for the comparison with model of physical indicators plus Framingham risk score and model of Framingham risk score.
